# Anti-counterfeiting SERS security labels derived from silver nanoparticles and aryl diazonium salts†

**DOI:** 10.1039/d2na00572g

**Published:** 2022-10-13

**Authors:** Da Li, Julien Brunie, Fan Sun, Philippe Nizard, Delphine Onidas, Aazdine Lamouri, Vincent Noël, Claire Mangeney, Giorgio Mattana, Yun Luo

**Affiliations:** Université Paris Cité, CNRS, Laboratoire de Chimie et de Biochimie Pharmacologiques et Toxicologiques F-75006 Paris France yun.luo@u-paris.fr; University Paris Cité, ITODYS, UMR 7086 75013 Paris France giorgio.mattana@univ-paris-diderot.fr; PSL Université, Chimie Paris Tech, IRCP, CNRS UMR 8247 75005 Paris France

## Abstract

The development of anti-counterfeiting inks based on surface-enhanced Raman scattering (SERS) labels have attracted great interest in recent years for their use as security labels in anti-counterfeiting applications. Indeed, they are promising alternatives to luminescent inks, which suffer from several limitations including emission peak overlap, toxicity and photobleaching. Most of the reported SERS security labels developed so far rely on the use of thiolate self-assembled monolayers (SAMs) for the immobilization of Raman reporters on metallic nanoparticle surface. However, SAMs are prone to spontaneous desorption and degradation under laser irradiation, thereby compromising the ink long-term stability. To overcome this issue, we develop herein a new generation of SERS security labels based on silver nanoparticles (Ag NPs) functionalized by aryl diazonium salts, carrying various substituents (–NO_2_, –CN, –CCH) with distinguishable Raman fingerprints. The resulting SERS tags were fully characterized by scanning electron microscopy (SEM), transmission electron microscopy (TEM), UV-vis absorption and SERS. Then, they were incorporated into ink formulations to be printed on polyethylene naphthalate (PEN) substrates, using handwriting or inkjet printing. Proof-of-concept Raman imaging experiments confirmed the remarkable potential of diazonium salt chemistry to design Ag NPs-based SERS security labels.

## Abstract 2

The development of anti-counterfeiting inks based on surface-enhanced Raman scattering (SERS) labels have attracted great interest in recent years for their use as security labels in anti-counterfeiting applications.

## Graphical abstract

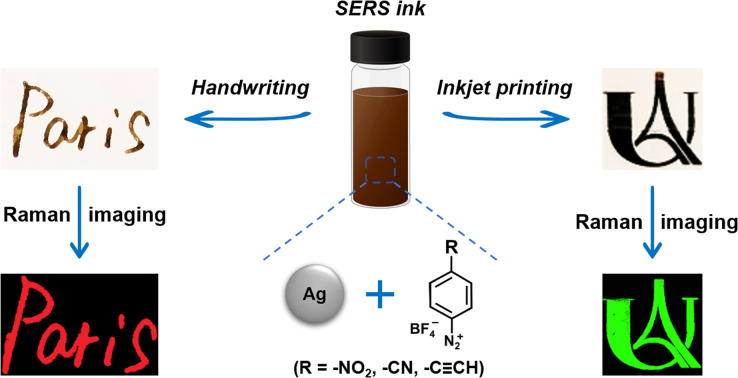

## Introduction

The development of surface-enhanced Raman scattering (SERS)^1^ labels has stimulated a wide interest in recent years for a large range of applications^2^ in biomedicine,^3^ sensors^4^ and security.^5^ In particular, few recent studies have demonstrated their potential as security labels for anti-counterfeiting technology,^6,7^ a priority research area for social economy and public safety. Indeed, it is estimated that the global economic value of counterfeiting and piracy could reach USD 4.2 trillion by 2022.^8^ To deal with this issue, several anti-counterfeiting ink formulations have been prepared, mainly based on luminescent labels,^9^ such as organic dyes, lanthanide doped nanomaterials, quantum dots (semiconductor and carbon based) or metal organic frameworks. Luminescent inks exhibit remarkable optical properties, which makes them easily visualized. Nevertheless, several limitations such as emission peak overlap, toxicity or photobleaching have been reported,^10^ compromising the long-term stability of the inks, their safe use and their coding capacity. Therefore, the design of innovative inks with narrow spectral linewidth, low toxicity and long-term chemical and photo-stability still remains an important challenge.

SERS labels can meet this challenge as they offer spectral signatures with multiple sets of narrow peaks, leading to low spectral overlap and large multiplexing capacity.^11,12^ In addition, multiple Raman labels can be excited *via* a single laser wavelength, with negligible photobleaching and high sensitivity provided by the strong electromagnetic field enhancement at the nanoparticles (NPs) vicinity. Despite these valuable properties, only few studies have reported the use of SERS labels for anti-counterfeiting applications and most of them rely on the use of thiolate self-assembled monolayers (SAMs)^13–15^ for the immobilization of the Raman labels on the plasmonic nanoparticles surface. However, the chemical stability of thiol-based SAMs is a critical issue.^16,17^ Indeed, it was shown that thiol-derived SAMs undergo spontaneous desorption in aqueous media after few days and are degraded under laser irradiation. Therefore, the development of alternative approaches fostering the robust grafting of Raman labels on the plasmonic NPs surface should lead to substantial improvements of the ink long-term stability. Aryl diazonium salts were shown over the past decade to be efficient surface modifiers for plasmonic nanoparticles,^18–22^ allowing fast surface grafting *via* robust metal–C covalent bonds. Moreover, these surface functionalization agents are easy to prepare from a large range of commercially available aniline derivatives, thereby providing a wide variety of functional groups. Thanks to these interesting characteristics, aryl diazonium salts were used to functionalize plasmonic NPs in order to obtain nanosensors,^23,24^ antimicrobial materials,^25^ contrast agents for Raman bioimaging^26^ or optical devices.^27–31^ But their use in anti-counterfeiting applications has never been reported yet, to the best of our knowledge.

**Fig. 1:**
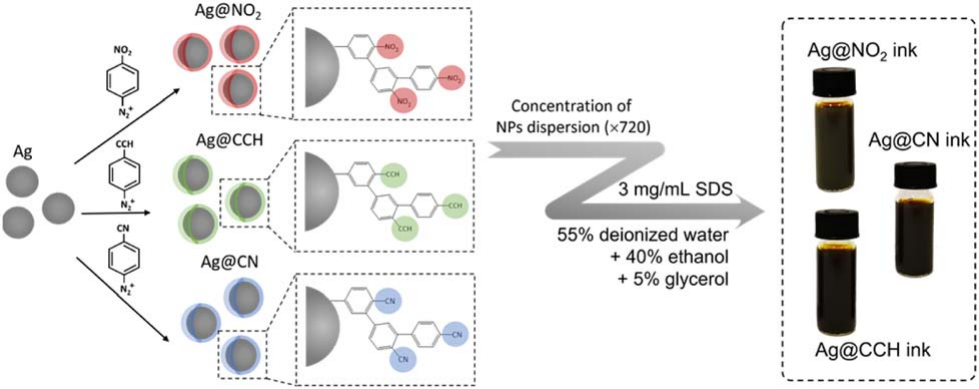
Illustration of the preparation process of SERS inks based on Ag NPs functionalized by aryl diazonium salts.

We fill this gap herein by designing new SERS security labels based on the combination of silver NPs and aryl diazonium salts. Three aryl diazonium salts with different *para*-substituents (–NO_2_, –CN, –CCH) were selected to functionalize Ag NPs. The choice of the diazonium salts was motivated by their characteristic distinguishable Raman fingerprints, which make them ideal candidates to act as Raman reporters (see Fig. 1). Indeed, the Raman signal of triple bond tags (–CN and –CCH) is located in the “Raman-silent” region (1750–2750 cm^−1^) while the –NO_2_ bond has a very strong characteristic peak at *ca.* 1330 cm^−1^.

The resulting nanohybrids Ag@NO_2_, Ag@CN and Ag@CCH, were fully characterized by scanning electron microscopy (SEM), transmission electron microscopy (TEM), UV-vis absorption and SERS. They were then successfully incorporated into ink formulations to be printed on polyethylene naphthalate (PEN) substrates, using hand-writing or inkjet printing, opening promising prospects for the development of a new generation of security labels.

## Experimental

### Synthesis of silver nanoparticles

Silver NPs were synthesized by the reduction of silver nitrate (≥99.0%, Sigma-Aldrich) following a previous report^32^ with appropriate modification. Typically, 240 μL of 0.1 M l-ascorbic acid (99%, Sigma-Aldrich) and 3.3 mL of 0.1 M sodium citrate (anhydrous, ≥99.5%, Sigma-Aldrich) were injected into a flask containing 240 mL of boiled deionized water under continuous stirring. After heating for one minute, 6 mL of 0.01 wt% polyvinylpyrrolidone (PVP, average molecular weight 360 000, Sigma-Aldrich) and 800 μL of 0.1 M aqueous silver nitrate were added to the flask sequentially. The heating and stirring were stopped after 5 minutes and a uniform orange solution was obtained. The solution was then cooled down to room temperature and stored in the fridge.

### Surface grafting of Raman reporters using aryl diazonium salts and 4-nitrothiolphenol

The surface functionalization of silver NPs was achieved by spontaneous grafting of diazonium salt at room temperature. Briefly, 1.2 mL of diazonium salt solution (1 mM for diazonium salts bearing –NO_2_ and –CN functional groups and 0.2 mM for diazonium salt bearing –CCH groups) was added to 240 mL silver solution. After 1 h of reaction, 4 mL of 1 wt% PVP was added to the solution, which was left to react for another 1 h. The obtained functionalized Ag NPs were washed by deionized water *via* 3 cycles of centrifugation/redispersion. The preparation of thiolate SAMs on AgNPs (as reference for stability test) was same. 200 μL of 4-nitrothiolphenol (1 mM) was mixed to 40 mL of AgNPs for 1 h, followed by another 1 h of reaction with 800 μL of 1 wt% PVP. Then 3 cycles of centrifugation/redispersion were performed as washing process.

### Preparation of the SERS inks

To prepare the SERS inks, 720 mL of functionalized Ag NPs solutions were concentrated to 1 mL and then added to 4 mL of a solution containing 55% deionized water, 40% ethanol and 5% glycerol. After vortexing the solution vigorously for 10 minutes, 15 mg of sodium dodecyl sulfate (≥99.0%, Sigma-Aldrich) was added to the ink solutions to tune their surface tension. The resulting solutions were then vigorously vortexed for 30 min to make NPs dispersed evenly.

### Inkjet printing

Inkjet-printing was performed using a Dimatix Materials Printer DMP-2850 (FUJIFILM Dimatix, Inc.). The cartridges used for the ink deposition were the 10 pL DMC-11610. Printing was performed using the standard Dimatix waveform (5 kHz) with a drop-spacing of 20 μm. During printing, the platen was heated up to 40 °C while the cartridge temperature was left at room temperature. After printing, samples were left to dry at ambient conditions.

### Characterization techniques

Morphological investigation of bare and functionalized Ag NPs was performed with transmission electron microscopy (TEM) using a Tecnai microscope (120 kV) and 4k × 4k Eagle camera (Thermofisher, USA). A Zeiss Supra 35 scanning electron microscope (SEM) with field-emission gun (FEG) equipped with scanning transmission electron microscopy (STEM) detector was employed to characterize the distribution of nanoparticles dispersed on copper grid with holely carbon film. The same samples were observed again in Jeol 2100plus transmission electron microscope (TEM) operated at 200 kV to observe the details of individual nanoparticle using double-tilt sample holder and Gatan Rio16 camera with 4k × 4k resolution. The extinction spectra of Ag colloidal solutions were recorded by UV-vis spectroscopy (Shimadzu 2700 UV-vis spectrometer). The hydrodynamic size were measured by dynamic light scattering (DLS) (CORDOUAN Visco Kin). The surface tension and viscosity of the SERS inks were studied by AquaPi portable tensiometer and viscometer DV-II (BROOKFIELD), respectively. The Raman spectra and images were obtained using a Horiba XploRA PLUS Raman microscope equipped with a 638 nm laser.

## Results and discussion

**Fig. 2:**
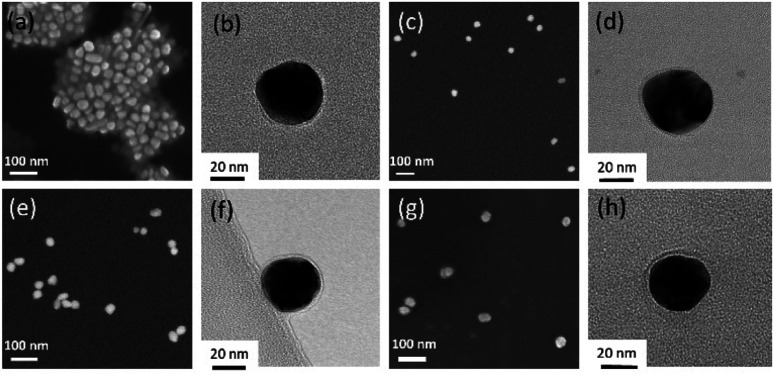
(a), (c), (e) and (g) SEM and (b), (d), (f) and (h) TEM images of (a) and (b) pristine Ag NPs, (c) and (d) Ag@NO_2_, (e) and (f) Ag@CCH and (g) and (h) Ag@CN NPs samples, respectively.

Spherical Ag NPs were synthesized by reduction of AgNO_3_ using l-ascorbic acid and sodium citrate. Based on SEM images (Fig. 2), the average diameter of the prepared Ag NPs was 25.3 nm (standard deviation *σ*_SEM_ = 3.8 nm, particle size distribution curves are shown in Fig. S1†). In order to create a variety of SERS labels, aryl diazonium salts with different *para*-functional groups (–NO_2_, –CCH and –CN) were used for the surface functionalization of Ag NPs. The surface grafting was based on a simple process involving the addition of diazonium salts to Ag NPs aqueous dispersions ([Ag NPs] = 3.7 × 10^−10^ M) at room temperature, in the presence of air. The TEM images recorded after the reaction between the Ag NPs and the diazonium salts (Fig. 2c–h and S2†) exhibit no obvious NPs aggregation and the mean particle size appears unchanged (*cf.* Fig. S1†), showing that the colloidal stability is preserved. In addition, a thin layer can be observed at the surface of the NPs after surface grafting, which could be assigned to the presence of polyaryl coating (Fig. 2d, f and h). The hydrodynamic size of Ag NPs was analyzed by dynamic light scattering (DLS), *cf.* Fig. S3,† where the size is increased of *ca.* 3–6 nm after the grafting of aryl diazonium salts. Indeed, the surface grafting reaction with diazonium salts involves the formation of aryl radicals, which can bind to both the Ag NP surface and to the already grafted aromatic groups through homolytic aromatic substitution, leading to polyaryl layers (as illustrated in Fig. 1) and resulting in increased hydrodynamic size.

**Fig. 3:**
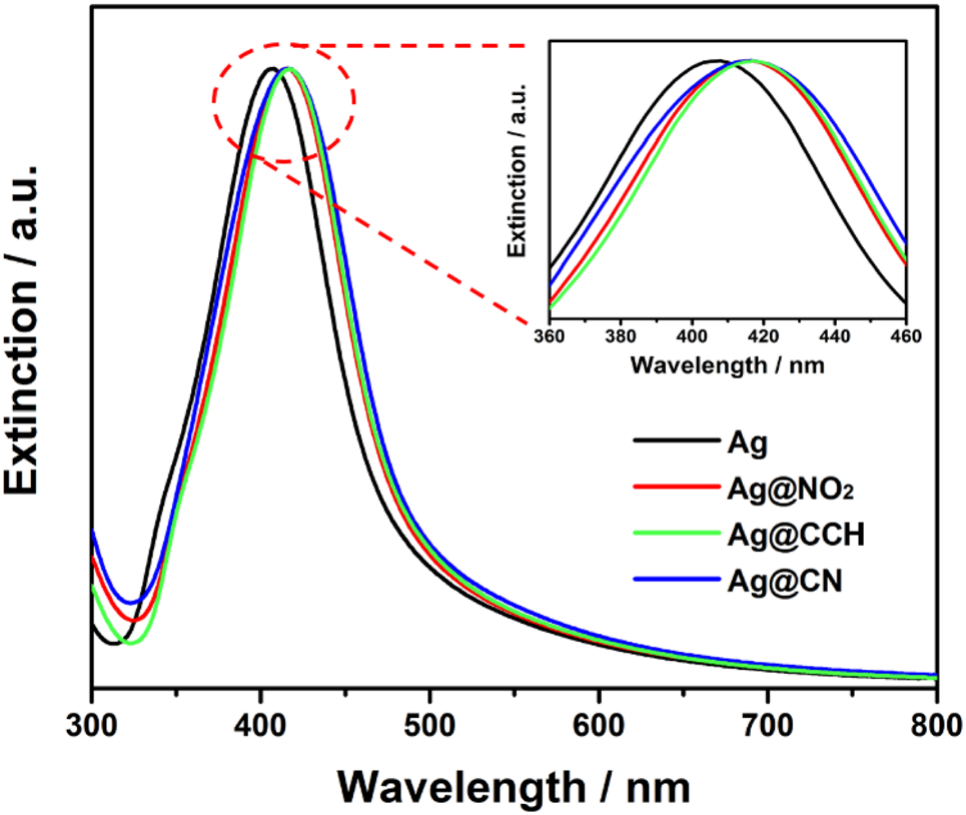
Normalized extinction spectra of Ag, Ag@NO_2_, Ag@CCH and Ag@CN NPs.

The extinction spectra of the pristine and functionalized Ag NPs (Fig. 3) were recorded to evaluate the effect of surface grafting on their optical properties. It appeared that the initial shape of the Ag NPs extinction profile remained broadly unchanged after functionalization confirming the stability of the colloidal dispersion. Interestingly, compared to pristine Ag NPs, the extinction spectra of Ag@NO_2_, Ag@CCH and Ag@CN NPs exhibit red shifts (7–9 nm), which can be explained by the modification of the local dielectric environment around the Ag NPs^33,34^ due to the polyaryl coating.

The hydrophilic SERS inks were further prepared by mixing concentrated aqueous dispersions of functionalized Ag NPs (3.7 × 10^−8^ M) with a solution containing 55% deionized water, 40% ethanol and 5% glycerol and adding afterwards SDS (3 mg mL^−1^) in order to reduce surface tension and improve writing and printing performance (*cf.*Fig. 1). Depending on the chemical groups carried by the functionalized Ag NPs, *i.e.* –NO_2_, –CCH or –CN, the corresponding inks were named Ag@NO_2_ ink, Ag@CCH ink and Ag@CN ink, respectively. The final surface tension and viscosity of SERS inks measured at room temperature were found to be 31.45 mN m^−1^ and 2.72 cP, respectively.

**Fig. 4:**
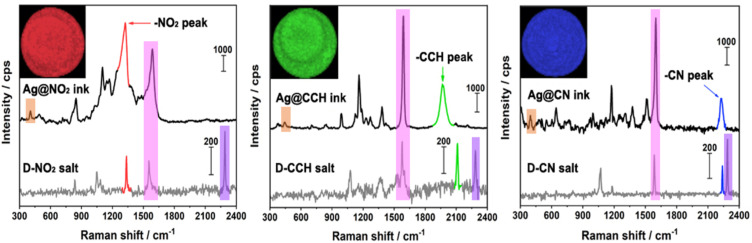
SERS spectra of the (a) Ag@NO_2_, (b) Ag@CCH and (c) Ag@CN inks deposited as dried drops on glass plates (excitation wavelength *λ* = 638 nm and exposure time was 2 s). The concentration of the aqueous diazonium salt solutions used for Ag NP surface functionalization was 1 mM for those with –NO_2_ and –CN groups and 0.2 mM for that with –CCH group. The corresponding Raman spectra of the parent aryl diazonium salts are displayed in grey lines. The Ag–C, aryl and –N_2_^+^ peaks are marked with orange, magenta and purple color, respectively. The insets show the Raman images of the corresponding ink dry drops (*λ* = 638 nm and exposure time was 0.2 s).

The SERS signatures of the various inks, deposited as dried drops on a glass plate, were then recorded using a 638 nm laser source (see Fig. 4). After surface grafting, the N≡N stretching vibration of the free aryl diazonium salts is no more detected at *ca.* 2280–2300 cm^−1^ while the vibrational fingerprints of the functional polyaryl layers surrounding the Ag NPs appear, including the aryl ring stretching vibration at *ca.* 1590–1600 cm^−1^ and the Ag–C stretching at *ca.* 395–405 cm^−1^.^19,35,36^ These characteristic features confirm the covalent binding of the organic layers derived from aryl diazonium salts on the surface of Ag NPs and the release of N_2_.^19^ Regarding the Raman reporter groups, they were detected *via* the presence of narrow and distinct peaks specific for each ink: *ν*_NO_2__ at 1327 cm^−1^ for Ag@NO_2_ ink, *ν*_CC_ at 1976 cm^−1^ for Ag@CCH ink and *ν*_CN_ at 2222 cm^−1^ for Ag@CN ink. The signal of –CCH in Ag@CCH is shifted compared to the diazonium precursor, probably due to the interaction between –CCH groups and the surface of Ag NPs, as reported previously.^37^ It is noteworthy that the Raman signals of the SERS labels (Ag@NO_2_, Ag@CCH, Ag@CN) were almost unchanged after their dispersion within the inks (see Fig. S4†).

Each SERS fingerprint was associated to a distinct color, as illustrated in the insets of Fig. 4 showing the Raman images of the various ink dried drops: the vibrational signature of Ag@NO_2_ ink has been assigned here to the red while the ones of Ag@CCH and Ag@CN inks were associated to the green and blue, respectively. Interestingly, the mixture of two types of labels within an ink allowed the creation of another SERS fingerprint arising from the combination of the different Raman signals, which could be associated to a new color code, as illustrated in Table 1. This strategy, consisting in mixing different labels within the same ink, allowed to enlarge the library of available SERS security labels. On the basis of the combination of the 3 Raman reporters, a total of 7 codes could be obtained experimentally with distinct spectral signatures. It is noteworthy that theoretically, the use of *n* kinds of Raman reporters should result in the generation of 2^*n*^ − 1 color codes.

**Table 1:** Color coding system of the SERS inks based on Ag NPs functionalized by aryl diazonium salts carrying –NO_2_, –CCH or CN groups. The right-hand side of the table displays the corresponding SERS spectra, recorded in the spectral regions of interest

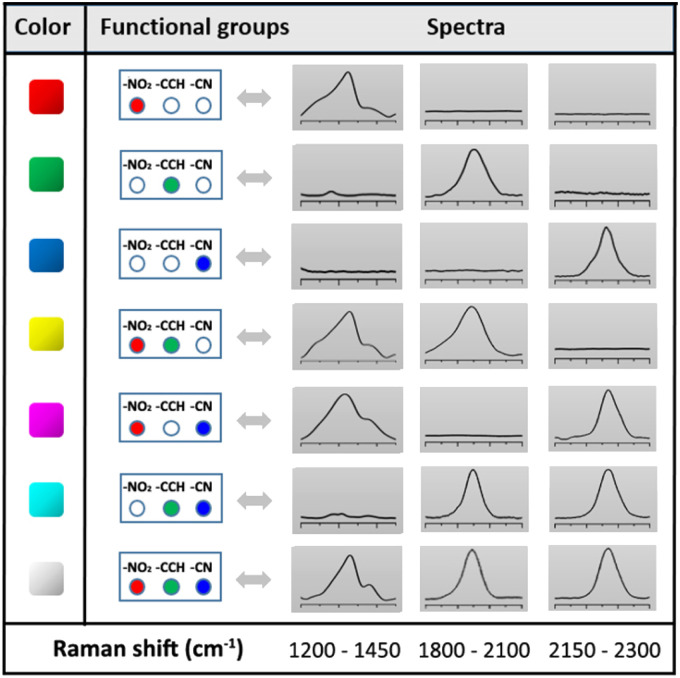

**Fig. 5:**
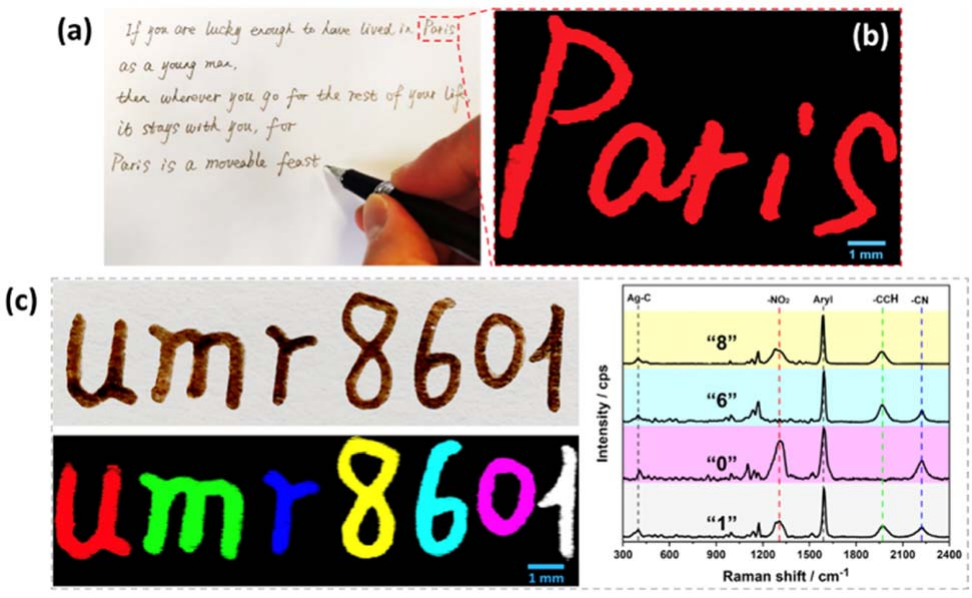
(a) Photograph of a famous sentence of E. Hemingway^38^ written using Ag@NO_2_ ink and (b) corresponding Raman image of the word “Paris”; (c) photograph of the writing of a laboratory name, UMR 8601 (top) and the corresponding Raman image (bottom). The pattern was written using the following inks: Ag@NO_2_ ink for letter “*u*”, Ag@CCH ink for “*m*”, Ag@CN ink for “*r*”, mixture of Ag@NO_2_ + Ag@CCH ink for “8”, mixture of Ag@CCH + Ag@CN ink for “6”, mixture of Ag@NO_2_ + Ag@CN ink for “0” and mixture of Ag@NO_2_ + Ag@CCH + Ag@CN ink for “1”. The corresponding Raman spectra of numbers 8, 6, 0 and 1 are displayed on the right part of the image. All SERS inks were loaded in a pen.

As a proof of concept for the use of these inks as SERS security labels, a pen was filled with the prepared SERS inks to write on paper. Fig. 5a shows a famous sentence of E. Hemingway,^38^ written using the diazonium salt-based SERS inks. The brown letters observed on simple optical images could be authenticated by Raman imaging, revealing an intense SERS signal arising from the ink used, here Ag@NO_2_ ink (see Raman image of the word Paris, see Fig. 5b). It is noteworthy that the SERS signals recorded on different area of written letters were very similar, emphasizing the homogeneity of the Raman signature (shown in Fig. S5†).

Interestingly, each letter could be written using a different ink, as illustrated in Fig. 5c on the laboratory name “UMR 8601” where each letter generates a different Raman signature associated to a distinct color code. The color-coding system can thus convert letters, visible to the naked eyes, into optical patterns with informative Raman signatures, only detectable by Raman techniques, thereby greatly increasing the difficulty of forgery.

**Fig. 6:**
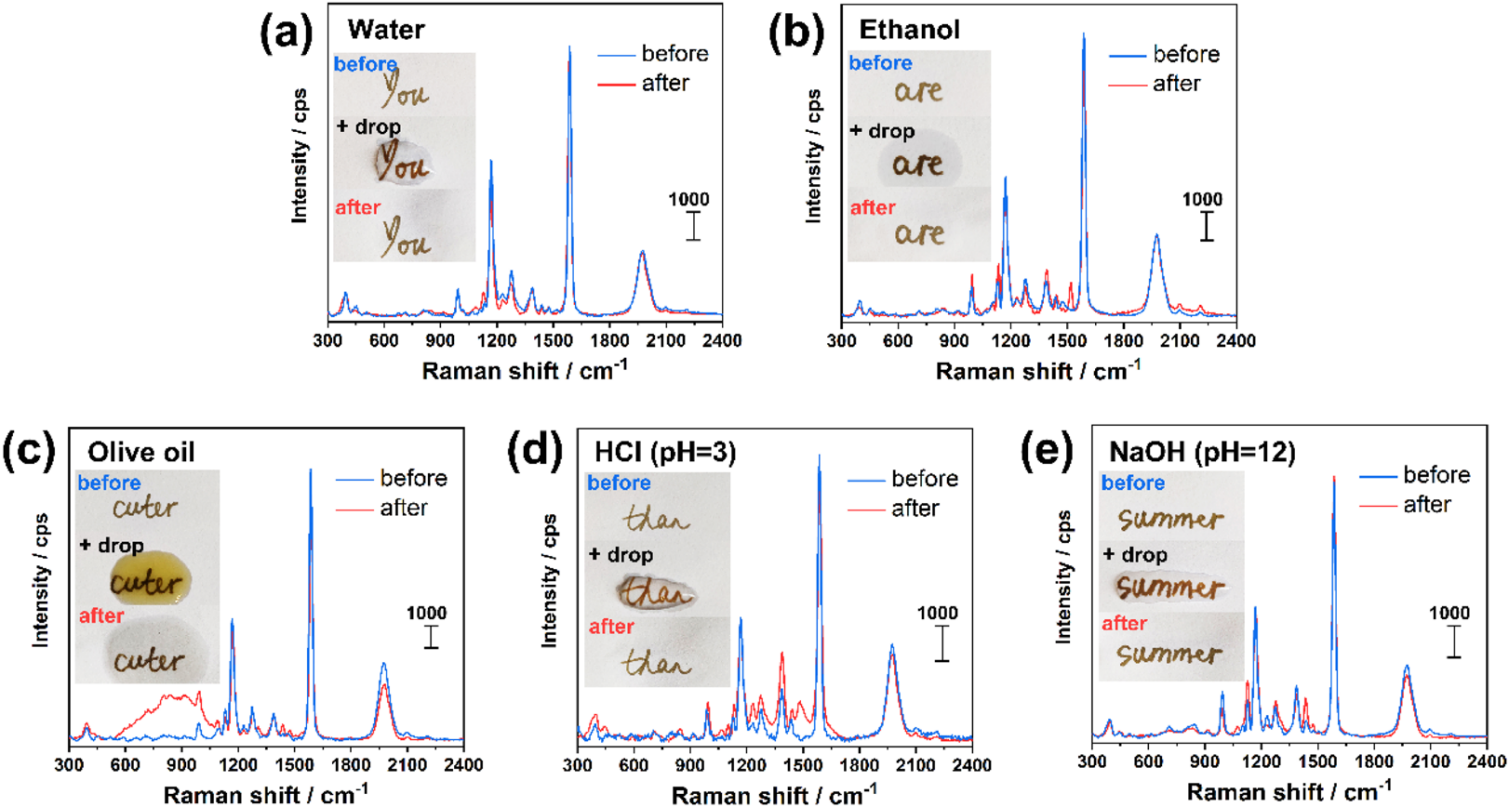
Investigation of the effect of different treatments on various words written using Ag@CCH ink on paper. (a) The word “you” was subjected to water deposition and drying; (b) the word “are” was subjected to ethanol deposition and drying; (c) the word “cuter” was subjected to olive oil deposition, and residue of oil was removed by paper napkin after 1 h; (d) the word “than” was subjected to acid (HCl at pH = 3) deposition and drying; (e) the word “summer” was subjected to alkaline solution (NaOH at pH = 12) deposition and drying. For each word, the SERS spectra were recorded before and after the corresponding treatment and drying (in air at room temperature).

To evaluate the stability of the inks once deposited on the substrate, the letters were subjected to various conditions, including irradiation under sunlight for over 5 months (Fig. S6†) and addition of a drop (100 μL) of water (Fig. 6a), ethanol (Fig. 6b), olive oil (Fig. 6c), acidic (HCl at pH = 3, Fig. 6d) and basic (NaOH at pH = 12, Fig. 6e) solution. Using Ag@CCH ink, the addition of a drop of water and ethanol (followed by drying in air) led to negligible change of the SERS signal intensity, whereas the treatments by olive oil, acid and base solution resulted in a decrease of –CCH (at 1976 cm^−1^) peak intensity, from 25% to 10%. It is worth noting that the modification of the SERS spectrum profile in the range of 600–1100 cm^−1^ before/after oil treatment could be attributed to some residue of olive oil remained adsorbed on the surface (Fig. 6c). Indeed, the presence of a broad luminescence peak (Fig. S7†) is consistent with previous Raman analysis on olive oil performed using a 633 nm laser.^39^ Remarkably, the prepared SERS inks also showed a high stability over time with an average signal decrease of only 9% after 5 months exposed to air (Fig. S6†). Ag@CN and Ag@NO_2_ inks also show excellent stability, as shown in Fig. S8.† The –CN (at 2222 cm^−1^) and –NO_2_ (at 1327 cm^−1^) peak intensity remain unchanged after the test by water and ethanol, and exhibit a slight decrease of 22–18% (for –CN peak) and 19–13% (for –NO_2_ peak) after oil, acid and basic solution treatments. It is important to note that, using conventional thiol-based coupling agent (*e.g.* 4-nitrothiolphenol), the resulted AgNPs ink (noted as Ag@SNO_2_, *cf.* experimental section for preparation details) is stable in water and ethanol treatment, and exhibits a comparable stability in oil treatment with a decrease of –NO_2_ peak intensity of 23%, *cf.* Fig. S8.† However, the Ag@SNO_2_ ink is unstable in contact to acid and basic solutions with a decrease of the –NO_2_ peak of 98–95%. These facts prove the superior stability of AgNPs inks derived from aryl diazonium salts compared to those functionalized by thiolate SAMs. The photostability of Ag@SNO_2_, Ag@NO_2_, Ag@CN, Ag@CCH inks was assessed by continuous laser exposure under Raman microscope. Each sample was irradiated for 0, 1, 3, 5 and 10 min. In the case of Ag@SNO_2_ inks, the recorded SERS spectra appear significantly modified after laser exposure with the progressive appearance of a set of new peaks (see Fig. S9a†) in the range of 1100–1500 cm^−1^, which can be related to the plasmon-driven formation of *p*,*p*′-dimercaptoazobenzene.^40^ In contrast, for Ag@NO_2_, Ag@CN and Ag@CCH inks, the spectra shape is barely changed upon laser irradiation, although the characteristic peak intensities are reduced of 18–32%. These results demonstrate that the structural information of diazonium salts derived inks is much more stable than that of Ag@SNO_2_ ink, which is a key parameter for the design of taggants.

**Fig. 7:**
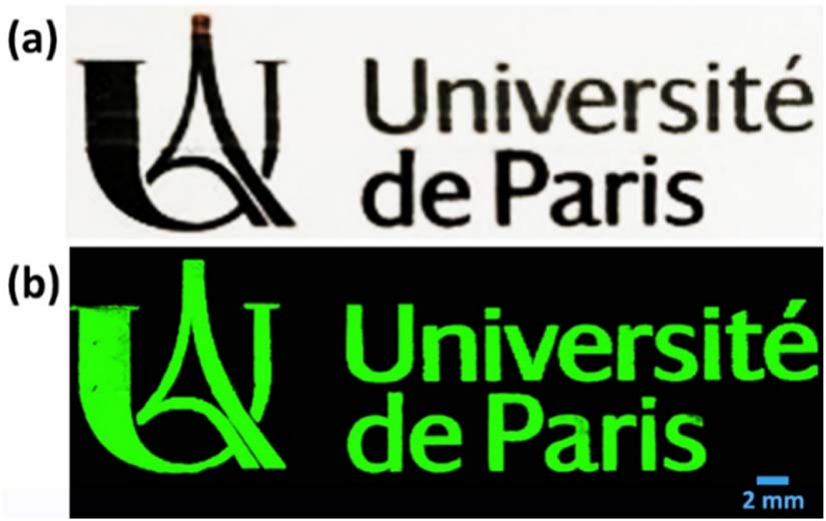
(a) Inkjet printing pattern of Ag@CCH ink (optical image) and (b) corresponding Raman image recorded by Raman microscope.

The deposition approach based on inkjet-printing was then investigated. Inkjet-printing is, indeed, particularly interesting for the prospective industrial utilization of the aforementioned inks. Inkjet-printing is a non-contact, digital deposition technique that can be easily adapted to any size of production scale: from rapid prototyping to small substrates to large-scale, large-area industrial production.^41^ Inkjet printing of a matrix of drops allowed the calculation of the average drop diameter (roughly 40 μm), which permitted the selection of the drop-spacing at 20 μm. Ink printability was rather good and allowed the reproduction of quite complex layouts such as the logo and the name of “Université de Paris” (see Fig. 7).

So far, inkjet-printing has already been employed for the fabrication of SERS-active substrates.^42,43^ Two approaches are commonly reported in the literature. A first approach is based on the deposition of inks made of metallic nanoparticles (Au, Ag) suspensions^44–46^ which, after sintering, allow the formation of conductive layers. A second approach consists in printing metallic salts (for instance AgNO_3_) on the surface of electrodes which are subsequently used for the electrochemical reduction of metallic cations into metallic nanoparticles.^47^ It is worth noting that, in both cases, the deposition of the Raman labels on the surface of the conductive pattern occurs after the metallic nanoparticles have already been deposited on the substrate. To the best of our knowledge, our paper therefore represents the first example of inkjet-printing deposition of already SERS-active nanotags.

## Conclusions

Ag NPs modified by aryl diazonium salts carrying various Raman reporter groups (–NO_2_, –CN, –CCH) were proposed as a new generation of SERS encoded-nanoparticles for anticounterfeiting applications. Compared to the commonly used surface functionalization strategy based on thiolate self-assembled monolayers, this approach includes the following advantages: (i) formation of robust covalent bonds between the Ag NP surface and the Raman reporters, a key parameter for ink chemical stability, (ii) simple grafting process through a fast and user-friendly protocol (in water at room temperature, in the presence of air). The hybrid NPs were successfully incorporated into ink formulations and printed on PEN substrates, *via* either handwriting or inkjet printing. Whatever the nature of the ink used, the formed patterns displayed homogeneous colors, visible to the naked eye. However, they exhibited distinct SERS signatures, depending on the type of functional groups carried by the Ag-based SERS tags which could only be detectable by Raman techniques, opening new avenues to improve anti-counterfeiting technologies. Moreover, the possibility to mix several diazonium-modified NPs carrying different Raman reporter groups was shown to provide new and distinct Raman spectra, thereby enlarging the library of available spectral combinations. The ink formulation showed excellent printability by inkjet-printing; this result, combined with the long stability at ambient conditions of the patterns, is suggestive of potential industrial large-scale production of security labels and tags for a large variety of objects.

## Conflicts of interest

The authors declare no competing financial interests.

## Supplementary Material

NA-004-D2NA00572G-s001
