# Supplementary material for: Anti-counterfeiting SERS security labels derived from silver nanoparticles and aryl diazonium salts
Source: Nanoscale Adv. 2022 Oct 13;4(23):5037–43. doi: 10.1039/d2na00572g (PMC9680943; doi:10.1039/d2na00572g)
Supplement: NA-004-D2NA00572G-s001 [file NA-004-D2NA00572G-s001.pdf]

Electronic Supplementary Material (ESI) for Nanoscale Advances.
This journal is © The Royal Society of Chemistry 2022

Supporting Information

Anti-Counterfeiting SERS Security Labels Derived from Silver Nanoparticles and Aryl Diazonium Salts

Da Li,[a] Julien Brunie,[b] Fan Sun,[c] Philippe Nizard,[a] Delphine Onidas,[a] Aazdine Lamouri,[b] Vincent Noël,[b] Claire Mangeney,[a] Giorgio Mattana,*[b] Yun Luo*[a]

[a] Université Paris Cité, CNRS, Laboratoire de Chimie et de Biochimie Pharmacologiques et Toxicologiques, F-75006 Paris, France

[b] University Paris Cité, ITODYS, UMR 7086, 75013 Paris (France)

[c] PSL Université, Chimie Paris Tech, IRCP, CNRS UMR 8247, 75005 Paris (France)

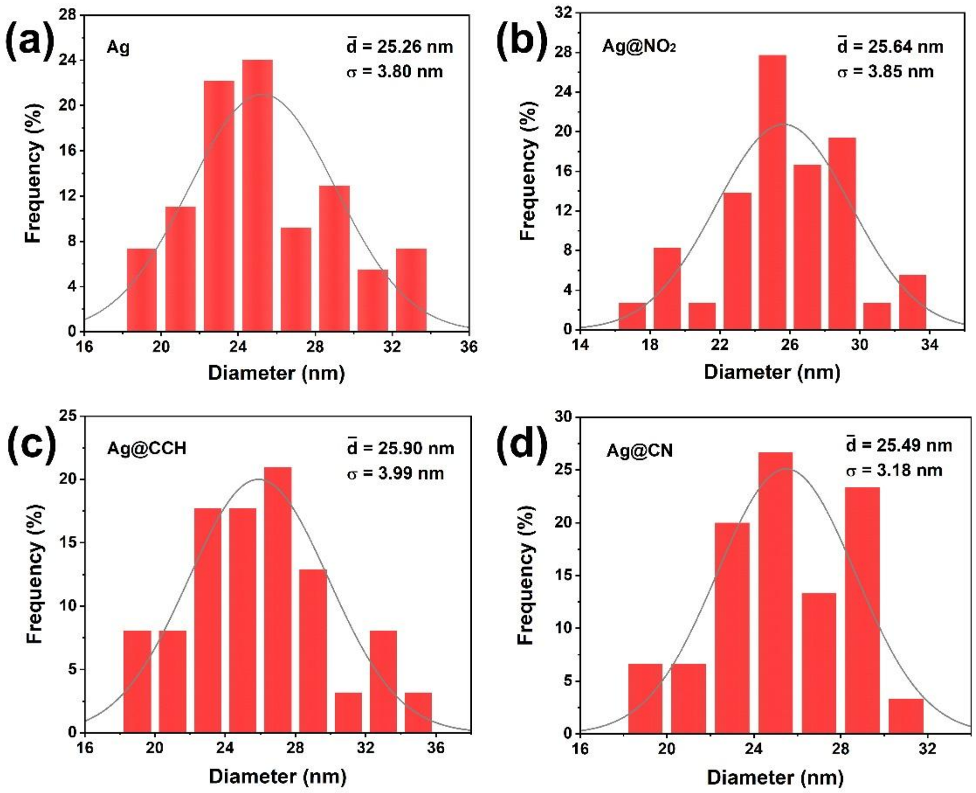

Fig. S1 Size distribution histograms measured from SEM images and associated distribution curves of (a) pristine Ag NPs, (b) Ag@NO_2, (c) Ag@CCH and (d) Ag@CN NPs.

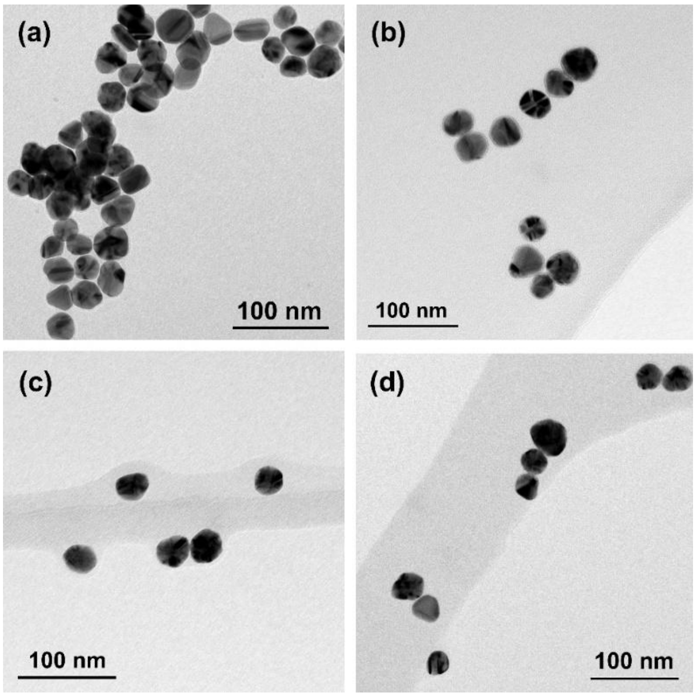

Fig. S2 TEM images of (a) pristine Ag NPs, (b) Ag@NO_2, (c) Ag@CCH and (d) Ag@CN NPs.

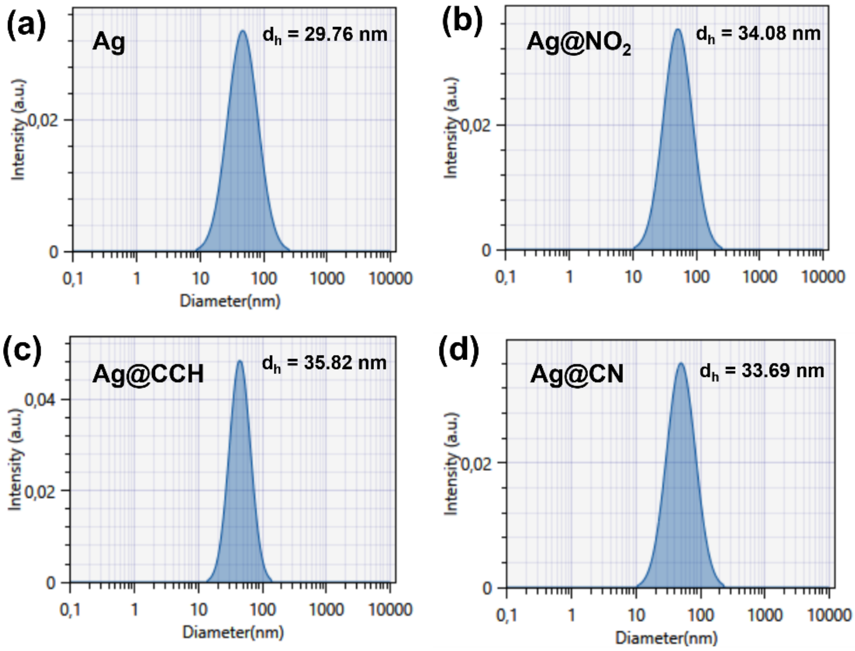

Fig. S3 The hydrodynamic size (d_h) from DLS measurements for (a) Ag, (b) Ag@NO_2, (c) Ag@CCH and (d) Ag@CN NPs.

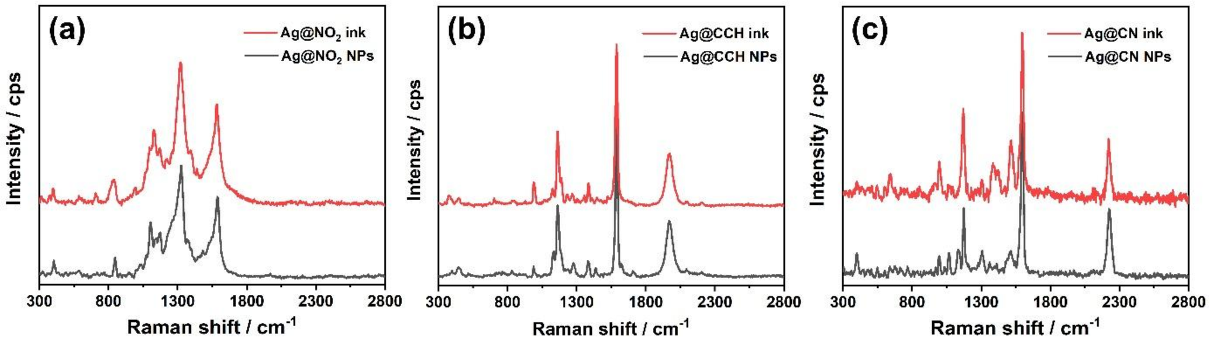

Fig. S4 SERS signals of (a) Ag@NO_2, (b) Ag@CCH and (c) Ag@CN before (black curves) and after dispersion in the inks (red curves).

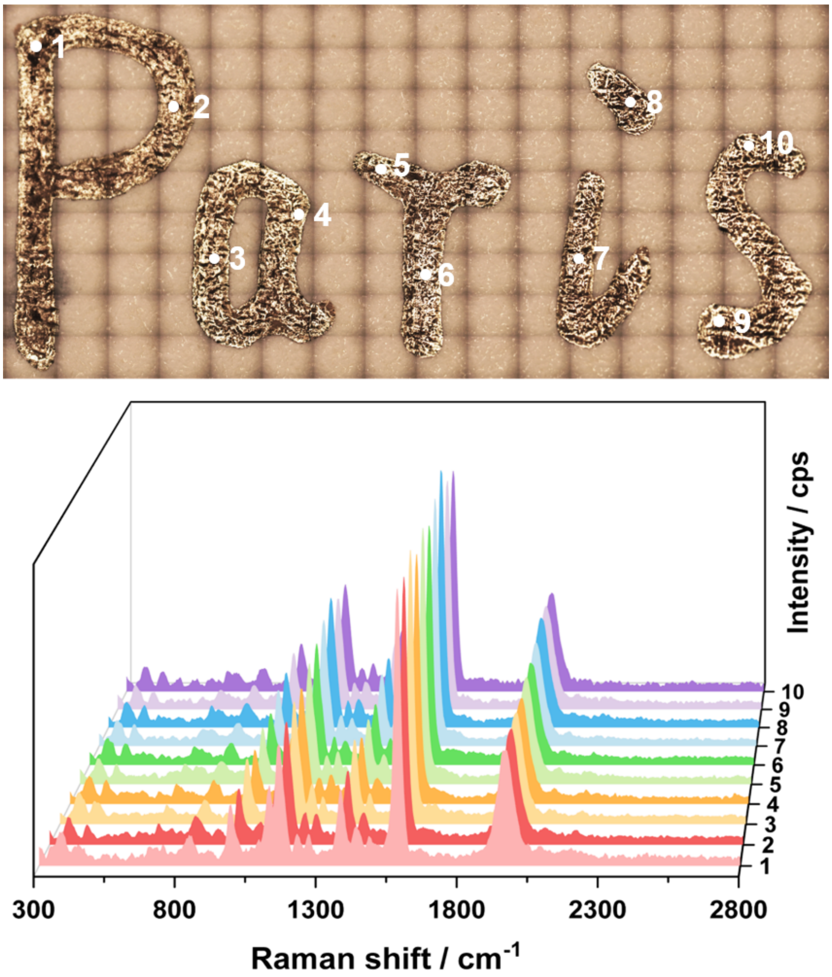

Fig. S5 SERS spectra collected from different spots of the handwritten word “Paris” (using Ag@CCH ink).

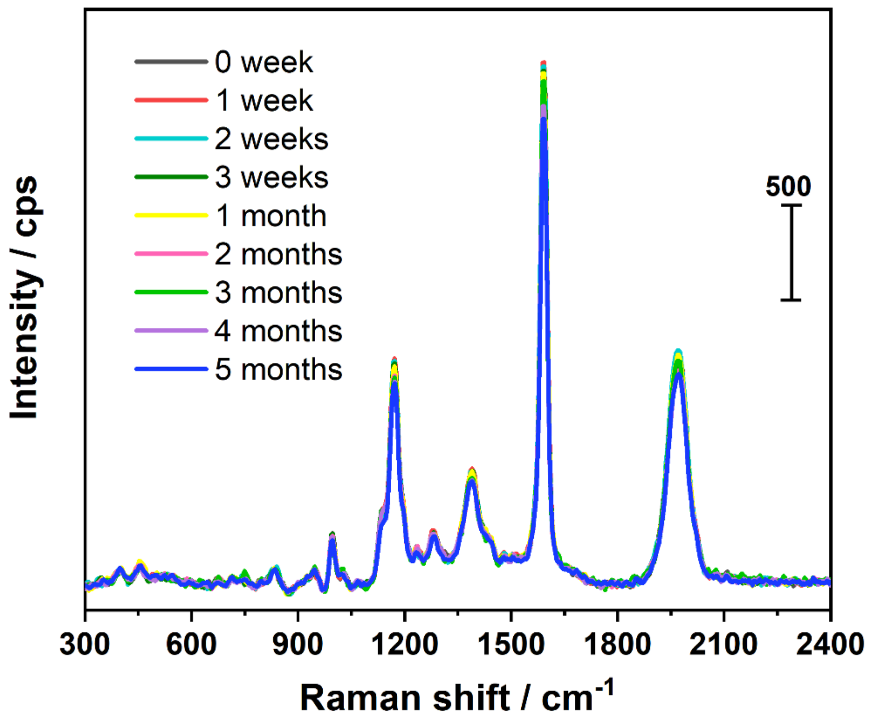

Fig. S6 Stability over time of Ag@CCH ink within 5 months.

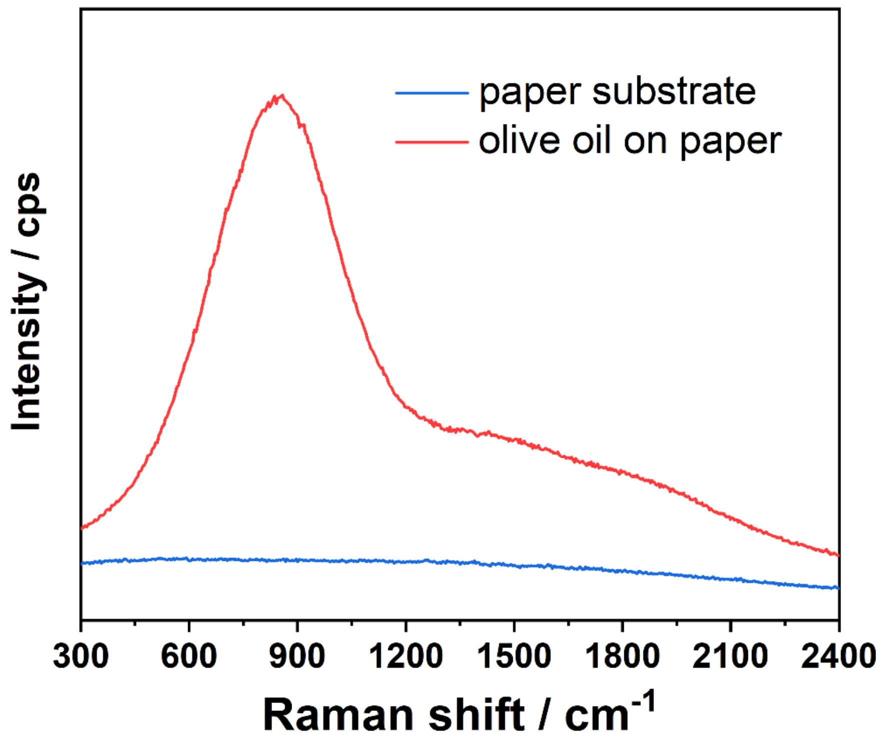

Fig. S7 Raman spectra of olive oil and the paper substrate under excitation of 638 nm laser.

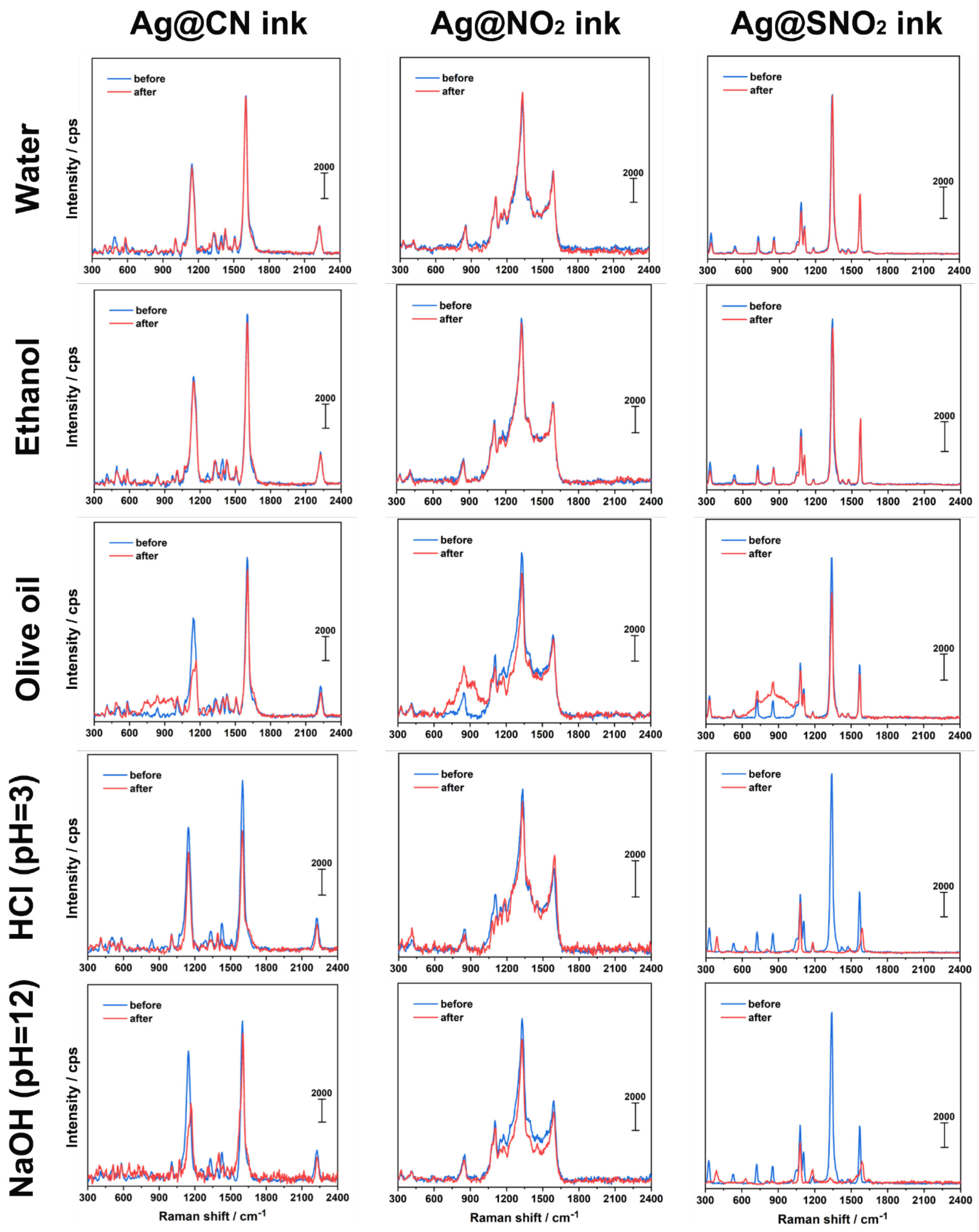

Fig. S8 Investigation of the stability of Ag@CN, Ag@NO_2 and Ag@SNO_2 ink in different medium. The testing method is same to that of Ag@CCH ink illustrated in maintext.

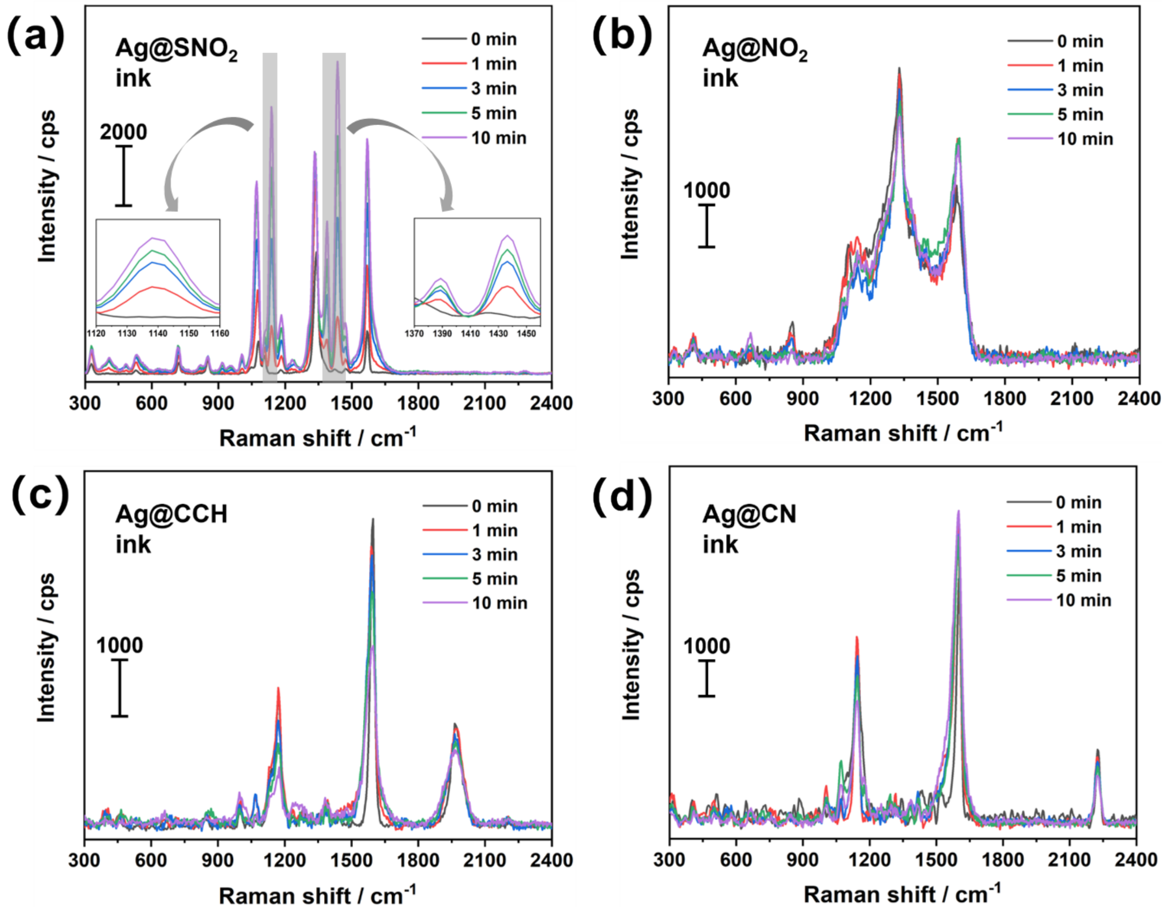

Figure S9. SERS spectra of (a) Ag@SNO_2, (b) Ag@NO_2, (c) Ag@CCH and (d) Ag@CN inks under laser irradiation for 0, 1, 3, 5 and 10 minutes, using 638 nm laser (10 mW) with objective 10x. The insets of Fig. (a) display a zoom of the time evolution of the peaks located in the shaded regions.
